# Integrative analyses of hub genes and their association with immune infiltration in adipose tissue, liver tissue and skeletal muscle of obese patients after bariatric surgery

**DOI:** 10.1080/21623945.2022.2060059

**Published:** 2022-04-12

**Authors:** Kemin Yan, Pengyuan Zhang, Jiewen Jin, Xin Chen, Hongyu Guan, Yanbing Li, Hai Li

**Affiliations:** Department of Endocrinology and Diabetes Center, The First Affiliated Hospital of Sun Yat-sen University, Guangzhou, Guangdong, China

**Keywords:** Obesity, bariatric surgery, adipose tissue, liver tissue, skeletal muscle, immune cell infiltration, bioinformatic analysis

## Abstract

Bariatric surgery (BS) is an effective treatment for obesity. Adipose tissue, liver tissue and skeletal muscle are important metabolic tissues. This study investigated hub genes and their association with immune infiltration in these metabolic tissues of obese patients after BS by bioinformatic analysis with Gene Expression Omnibus datasets. Differentially expressed genes (DEGs) were identified, and a protein–protein interaction network was constructed to identify hub genes. As a result, 121 common DEGs were identified and mainly enriched in cytokine–cytokine receptor interactions, chemokine signaling pathway, neutrophil activation and immune responses. Immune cell infiltration analysis showed that the abundance of M1 macrophages was significantly lower in adipose and liver tissue after BS (p<0.05). Ten hub genes (TYROBP, TLR8, FGR, NCF2, HCK, CCL2, LAPTM5, MNDA and S100A9) that were all downregulated after BS were also associated with immune cells. Consistently, results in the validated dataset showed that the expression levels of these hub genes were increased in obese patients and mice, and decreased after BS. In conclusion, this study analysed the potential immune and inflammatory mechanisms of BS in three key metabolic tissues of obese patients, and revealed hub genes associated with immune cell infiltration, thus providing potential targets for obesity treatment.

## Introduction

Obesity is one of the world’s largest health problems, and it has physical and psychosocial consequences. According to the World Health Organization (WHO), 39% of adults worldwide were overweight and 13% were obese in 2016 [1]. Moreover, obesity has been reported to substantially increase the risk of metabolic disease, cardiovascular disease, musculoskeletal disease, Alzheimer’s disease, depression and some types of cancer [2,3]. Furthermore, obesity might result in reduced life quality, increased unemployment, lower productivity and social disadvantages [2]. Bariatric surgery (BS) is an effective treatment for severe obesity and many obesity-related comorbid conditions [4,5]. The most common types of BS worldwide are laparoscopic sleeve gastrectomy, laparoscopic Roux-en-Y gastric bypass, laparoscopic adjustable gastric banding and duodenal switch [4]. However, the mechanisms by which BS affects obesity remain to be explored. Clarification of the mechanisms of BS may help to identify other treatment strategies for obesity.

Adipose tissue, liver tissue and skeletal muscle are important metabolic tissues that play important roles in the progression of insulin resistance and obesity [6]. Previous studies reported that hypertrophy, inflammatory response and adipokine dysregulation of adipose tissue may affect the outcomes of BS [7] and suggested that BS could reduce hepatic fat contents and improve hepatic insulin resistance [8]. In addition, Peter H Albers et al. demonstrated that the improved molecular insulin-sensitive phenotype of skeletal muscle and adipose tissue contributed to the improved whole body insulin action and substantial weight loss after BS [9]. Therefore, adipose tissue, liver tissue and skeletal muscle may also play critical roles in the beneficial effects of BS.

Immune cells have been shown to play an important role in the pathogenesis of obesity-related chronic diseases [10]. Tissue-associated immune cells in adipose and liver tissue, especially T-cell subpopulations, have been found to contribute to obesity and its comorbidities by promoting systemic inflammation [11]. Increasing evidence also suggests that increased immune cell infiltration in skeletal muscle may induce myocyte inflammation and contribute to insulin resistance in obesity [12]. Meanwhile, several changes have been reported to occur in adaptive immunity cells after bariatric surgery, including a reduction in T-cell counts and an increase in B regulatory cells [13]. The change in immune cell infiltration and differentially expressed immune-related genes in adipose tissue after BS have also been analysed by bioinformatics analysis [14], and the evidences showed that immune cells may play an important role in the beneficial effects of BS on the treatment of obesity and obesity-related comorbid conditions.

Therefore, the present study investigated hub genes and their association with immune infiltration in adipose tissue, liver tissue and skeletal muscle of obese patients after BS. This study may reveal the mechanisms by which BS reduces weight and improves metabolism from a new perspective based on immune regulation effects of BS.

## Methods

### Microarray data

The datasets GSE59034, GSE66921, GSE106737, GSE134913 and GSE5109, which include gene expression profiles generated from metabolic tissues of the patients before and after BS, were downloaded from the Gene Expression Omnibus (GEO) database (http://www.ncbi.nlm.nih.gov/geo). The datasets GSE59034 and GSE66921 included adipose tissue samples, GSE106737 included liver samples, and GSE134913 and GSE5109 included skeletal muscle samples. Detailed information on these included GEO datasets is displayed in Table 1.

**Table 1.** Detailed information on the included GEO datasets

Series	Platform	Analysis type	Tissue	Number of tissue samples from obese subjects		Title of study	Year	Reference
				Before BS	After BS			
GSE59034	GPL11532	Array	Adipose tissue	n = 16	n = 16	A Memory for Obesity in Adipose Tissue	2017	PMID: 30332637
GSE66921	GPL13607	Array	Adipose tissue	n = 4	n = 4	Transcriptional profiling of subcutaneous adipose tissue of morbidly obese subjects before and after 2 years of bariatric surgery	2017	NA
GSE106737	GPL16686	Array	Liver	n = 41	n = 41	Microarray analysis of liver biopsies at baseline and after one year follow up from 21 patients with histologically proven NASH (responders, bariatric surgery)	2019	PMID: 31701087
GSE134913	GPL26966	Array	Skeletal muscle	n = 14	n = 28	Dynamic changes of muscle insulin sensitivity after metabolic surgery I	2019	PMID: 31519890
GSE5109	GPL570	Array	Skeletal muscle	n = 3	n = 3	Gastric Bypass Human Obese Muscle	2006	PMID: 16849634

### Data preprocessing and identification of differentially expressed genes (DEGs)

**Figure 1.**
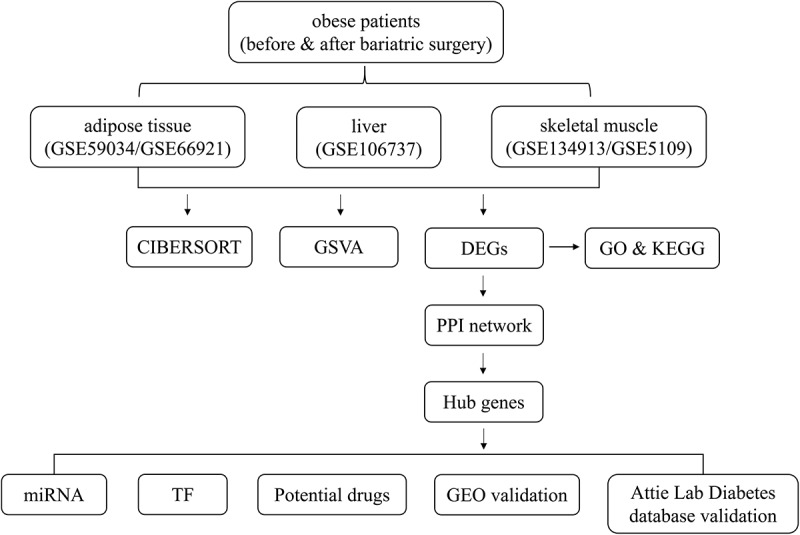
Flow chart of the study design.

The study design is shown in Figure 1. The expression matrices of the two datasets, GSE59034 and GSE66921, were combined. The expression matrices of GSE134913 and GSE5109 were also combined. Then, batch normalization was conducted using the ‘sva’ R package. A quantile–quantile plot (Q–Q plot) was applied to visualize the effect of removing inter-batch differences (**Supplemental Figure 1**). A total of 9884 genes expressed in all three metabolic tissues were selected for the next analysis. The ‘limma’ R package was applied to identify DEGs between the samples before and after BS in adipose tissue, liver tissue and skeletal muscle. Differences with *p* values<0.05 were considered statistically significant. A hierarchical clustering analysis was performed and volcano plot was generated by the ‘pheatmap’ and ‘ggplot2’ R packages, respectively. In the heatmap, only representative DEGs are shown.

### Enrichment analysis

The common DEGs were defined as the intersection of DEGs in at least two metabolic tissues. A Venn diagram of the DEGs was plotted by the ‘VennDiagram’ R package. Then, a Gene Ontology (GO) enrichment analysis and Kyoto Encyclopaedia of Genes and Genomes (KEGG) pathway analysis of the common DEGs were conducted using the ‘clusterProfiler’ R package. The cut-off criteria for both the KEGG and GO analysis was q-value<0.05. In addition, a gene set variation analysis (GSVA) was performed by the ‘GSVA’ R package to identify the pathway alterations in adipose tissue, liver tissue and skeletal muscle after BS. Pathways with *p*< 0.05 were considered statistically significant.

### Construction of a protein–protein interaction (PPI) network and identification of hub genes

The common DEGs were sent to STRING (http://www.string-db.org/) to construct the initial PPI network using a minimum required interaction score >0.4. The CytoHubba plugin in Cytoscape software was applied to identify key nodes in the PPI network. The top 10 genes screened by the degree method were identified as hub genes.

### Construction of target gene-transcription factor (TF) and target gene-miRNA networks and identification of potential drugs

The TFs of the hub genes were predicted using NetworkAnalyst (https://www.networkanalyst.ca/). The miRNAs of the hub genes were predicted using miRNet (https://www.mirnet.ca/). The target gene-TF and target gene-miRNA networks were visualized using Cytoscape software. The potential drugs or molecular compounds that interacted with the hub genes were predicted by searching the Drug Gene Interaction Database (DGIdb) (https://www.dgidb.org). The drug-gene interaction network was also visualized by Cytoscape software.

### Evaluation of immune cell infiltration and correlation analysis between hub genes and infiltrating immune cells

To further calculate the differences in the relative percentages of infiltrating immune cells after BS, the CIBERSORT algorithm was applied. The Wilcoxon test was applied to identify significant immune cell subpopulations, and *p*< 0.05 was considered statistically significant. Then, the rcorr () function of the ‘Hmisc’ R package was used to perform a Spearman correlation analysis on the hub genes and infiltrating immune cells. A value of *p*< 0.05 was considered statistically significant. The ‘corrplot’ R package was used to visualize the results.

### Validation of hub genes

The GEO dataset GSE72158, which includes data on adipose tissue samples of obese subjects before and after BS was included to verify the changes in the hub genes after BS. In addition, the expression changes of the hub genes in adipose tissue between obese and normal weight people were validated in the GEO dataset GSE151839. The Attie Lab Diabetes database (http://diabetes.wisc.edu) was also used to verify expression changes of hub genes in adipose tissue between obese and lean B6 mice. The Wilcoxon test was performed and *p*< 0.05 was considered statistically significant.

## Results

### Identification of common DEGs

**Figure 2.**
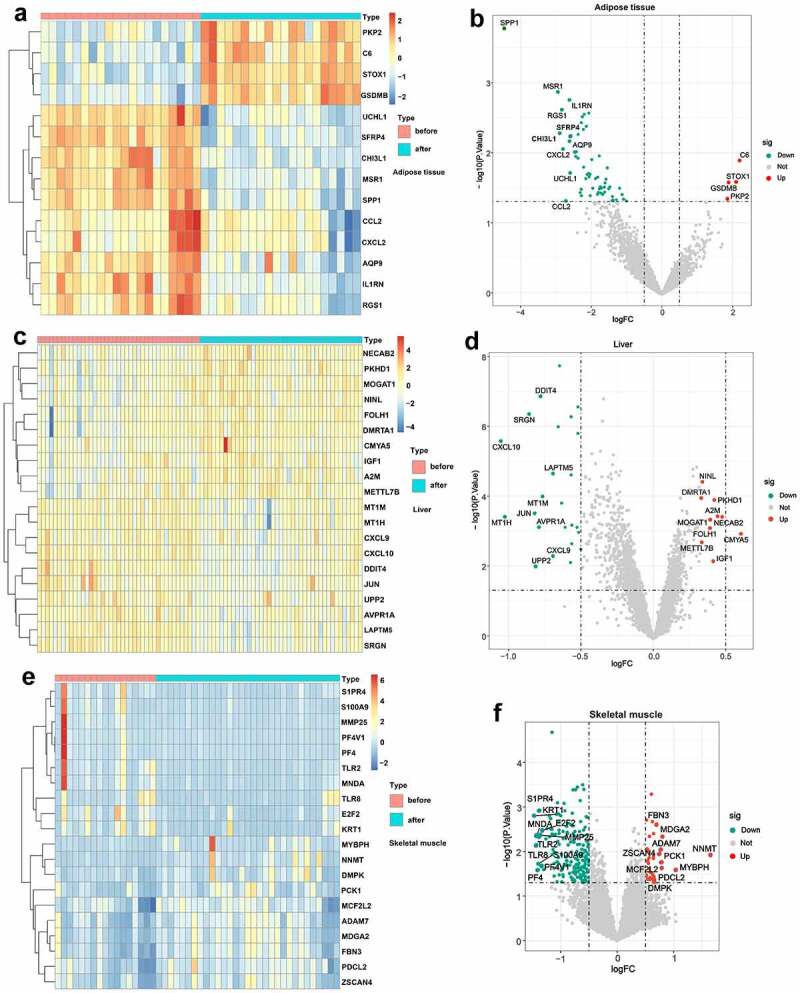
Visualization of DEGs in adipose tissue, liver tissue and skeletal muscle before and after BS. (a, c, e) Heatmap showing the expression of representative DEGs of each sample in adipose tissue (combination and normalization of GSE59034 and GSE66921), liver tissue (GSE106737) and skeletal muscle (combination and normalization of GSE134913 and GSE5109). The top 10 upregulated DEGs and top 10 downregulated DEGs ranked by logFC are shown as representative DEGs. In adipose tissue, only 4 upregulated DEGs were observed. (b, d, f,) DEGs in adipose tissue, liver tissue and skeletal muscle are presented in a volcano map. The representative DEGs were labelled with the gene names.

Based on the defined criteria, 64 DEGs were identified in adipose tissue after standardization of the GSE59034 and GSE66921 datasets. In total, 1190 DEGs were identified in liver tissue with the GSE106737 dataset. In addition, 369 DEGs were identified in skeletal muscle with the standardization of the GSE134913 and GSE5109 datasets. The representative DEGs in adipose tissue (Figure 2a, b), liver tissue (Figure 2c, d) and skeletal muscle (Figure 2e, f) are shown in the heatmaps and volcano plots.

### Enrichment analysis

**Figure 3.**
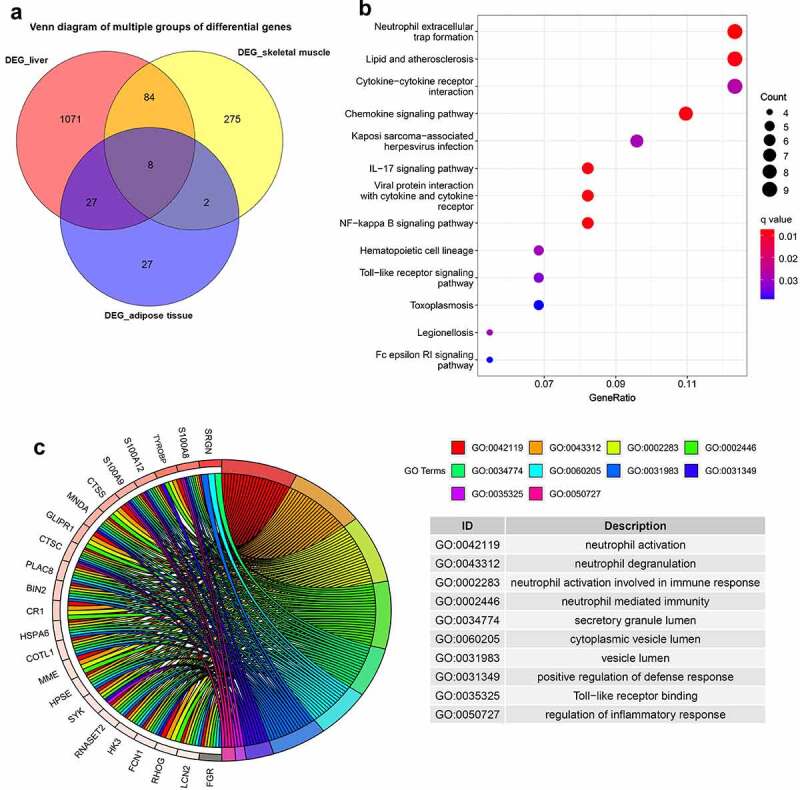
Venn diagram and enrichment analysis of DEGs from adipose tissue, liver tissue and skeletal muscle before and after BS.

As presented in Figure 3a, 121 common DEGs were identified, and they were defined as the intersection of DEGs in at least two of the three metabolic tissues. The KEGG enrichment analysis showed that these common DEGs were mainly involved in neutrophil extracellular trap formation, cytokine–cytokine receptor interaction, chemokine signalling pathway, IL-17 signalling pathway, NF-kappa B signalling pathway, Toll-like receptor signalling pathway and Fc epsilon RI signalling pathway (Figure 3b). The GO enrichment analysis showed that these genes were mainly enriched in neutrophil activation, immune response, positive regulation of defence response, Toll-like receptor binding and regulation of inflammatory response (Figure 3c).

(a) Venn diagram of DEGs in adipose tissue, liver tissue and skeletal muscle. The common DEGs were defined as the intersection of DEGs in at least two metabolic tissues. A total of 121 common DEGs were identified. (b) KEGG analysis of common DEGs. (c) GO analysis of common DEGs. The cut-off criteria for both the KEGG and GO analysis was q-value<0.05.

**Figure 4.**
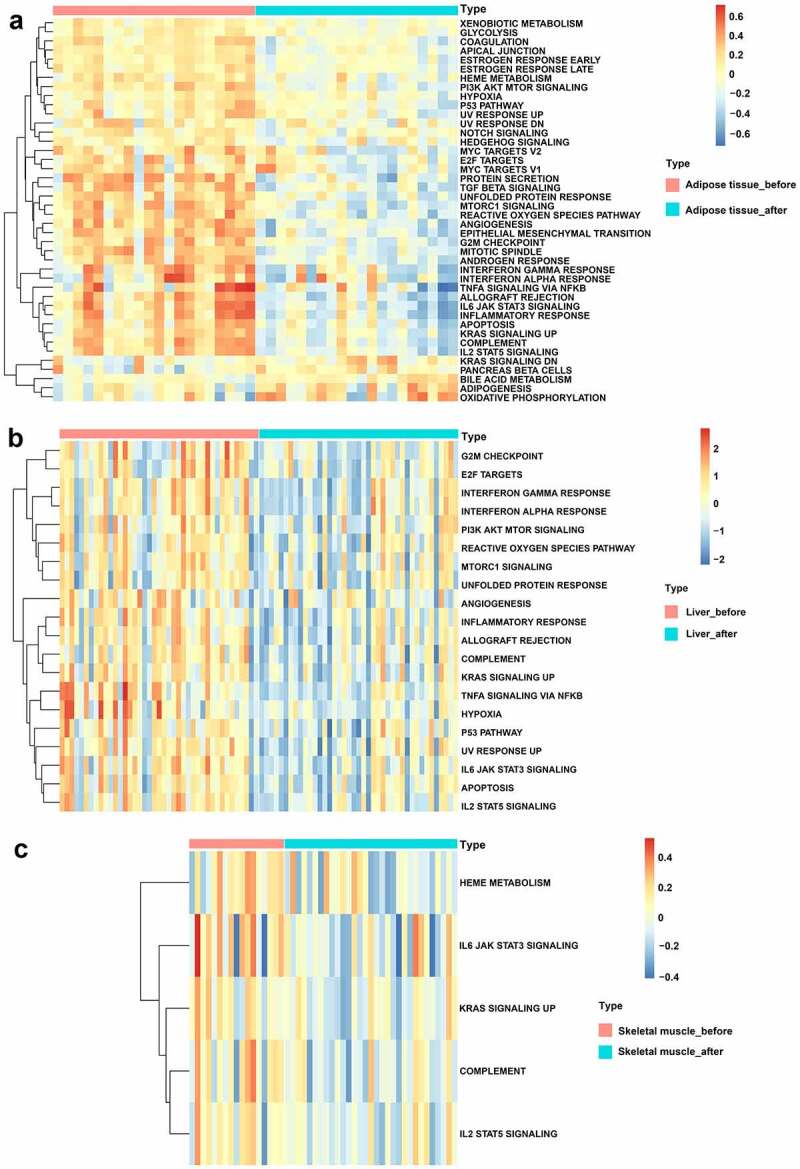
GSVA displaying pathway differences in adipose tissue, liver tissue and skeletal muscle before and after BS. GSVA was performed using the ‘GSVA’ R package to identify the pathway alterations in adipose tissue, liver tissue and skeletal muscle after BS. (a) Heatmap of GSVA in adipose tissue. (b) Heatmap of GSVA in liver tissue. (c) Heatmap of GSVA in skeletal muscle. Pathways with *p*< 0.05 were considered statistically significant.

Based on the ‘Hallmark’ gene sets, GSVA was performed to identify the possible mechanisms of BS effects. As displayed in Figure 4a, bile acid metabolism, adipogenesis and oxidative phosphorylation signalling pathways were upregulated while glycolysis, PI3K-AKT-MTOR signalling, P53 pathway, NOTCH signalling, HEDGEHOG signalling, TGF beta signalling, unfolded protein response, mTORC1 signalling, reactive oxygen species pathway, angiogenesis, IL6-JAK-STAT3 signalling, inflammatory response, apoptosis and IL2-STAT5 signalling pathways were downregulated in adipose tissue after BS. In addition, the PI3K-AKT-MTOR signalling, reactive oxygen species pathway, mTORC1 signalling, unfolded protein response, angiogenesis, inflammatory response, complement, hypoxia, P53 pathway, IL6-JAK-STAT3 signalling, apoptosis and IL2-STAT5 signalling pathways were also downregulated in liver tissue after BS (Figure 4b). As shown in Figure 4c, the IL6-JAK-STAT3 signalling, complement and IL2-STAT5 signalling pathways were also downregulated in skeletal muscle after BS.

### PPI network analysis and identification of hub genes

**Figure 5.**
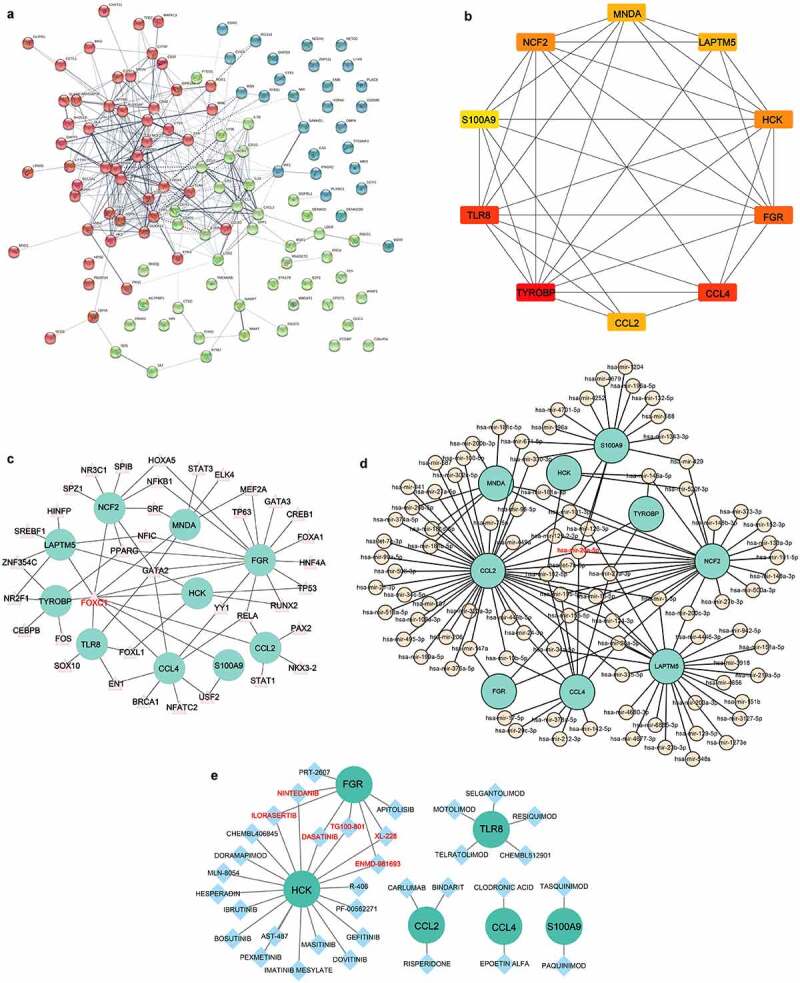
Identification of hub genes and prediction of TF, miRNA and drug-gene networks.(a) Construction of the PPI network of the 121 common DEGs. (b) Identification of hub genes by PPI network. (c) Construction of the hub gene-TF network. (d) Construction of hub genes-miRNA network. (e) Hub gene-drug interaction network.

The PPI network of 121 common DEGs was constructed based on STRING (Figure 5a). The top 10 hub genes selected by the degree method included transmembrane immune signalling adaptor TYROBP (TYROBP), C-C motif chemokine ligand 4 (CCL4), toll like receptor 8 (TLR8), FGR proto-oncogene, Src family tyrosine kinase (FGR), neutrophil cytosolic factor 2 (NCF2), HCK proto-oncogene, Src family tyrosine kinase (HCK), C-C motif chemokine ligand 2 (CCL2), lysosomal protein transmembrane 5 (LAPTM5), myeloid cell nuclear differentiation antigen (MNDA) and S100 calcium binding protein A9 (S100A9) (Figure 5b). These hub genes were all downregulated in the three metabolic tissues after BS.

### Construction of target gene-TF and target gene-miRNA networks and identification of potential drugs

In the hub gene-TF network, the top three targeted hub genes for TFs were FGR, which was modulated by 16 TFs; NCF2, which was modulated by 9 TFs; and CCL4, which was modulated by 8 TFs (Figure 5c). The TF FOXC1 may control all of the hub genes (Figure 5c). In addition, the top three targeted hub genes for miRNAs were CCL2, which was modulated by 43 miRNAs; LAPTM5, which was modulated by 25 miRNAs; and NCF2, which was modulated by 23 miRNAs (Figure 5d). The miRNA that may control the largest number of hub genes (five genes) was hsa-miR-20a-5p (Figure 5d). In the hub gene-drug interaction network, nintedanib, ilorasertib, dasatinib, TG100-801, ENMD-981693 and XL-228 were found to interact with two hub genes (FGR and HCK) (Figure 5e).

### Immune cell infiltration evaluation and correlation analysis between hub genes and infiltrating immune cells

**Figure 6.**
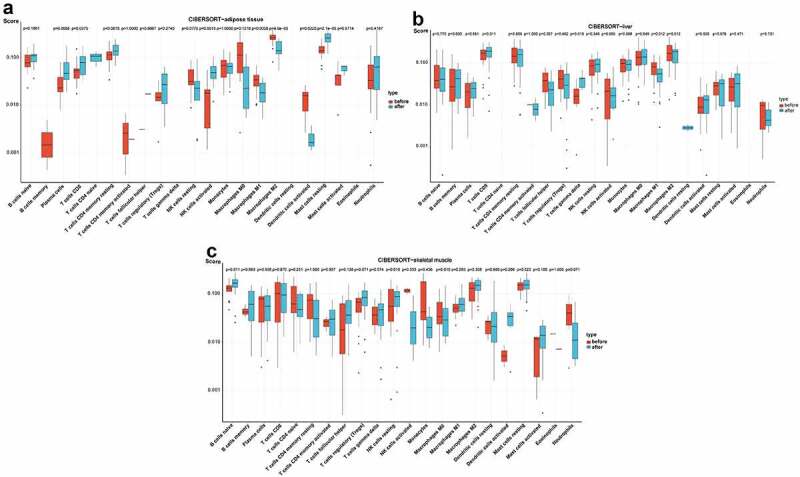
Analysis of immune cell infiltration in adipose tissue, liver tissue and skeletal muscle before and after BS according to the CIBERSORT algorithm. The differences in the relative percentages of infiltrating immune cells after BS were calculated by the CIBERSORT algorithm. The relative abundance of 22 different immune cells in (a) adipose tissue, (b) liver tissue and (c) skeletal muscle before and after BS. The Wilcoxon test was applied, and *p*< 0.05 was considered statistically significant.

In the box plot of the immune cell infiltration difference, the abundance of plasma cells, CD8 T cells, activated NK cells and resting mast cells was significantly higher in adipose tissue after BS, while the abundance of M1 macrophages, M2 macrophages and activated dendritic cells was significantly lower in adipose tissue after BS (*p*< 0.05, Figure 6a). As presented in Figure 6b, gamma delta T cells showed greater infiltration, while M1 macrophages showed less infiltration in the liver tissue after BS (*p*< 0.05). In skeletal muscle, the abundance of naive B cells was significantly higher after BS (*p*< 0.05, Figure 6c).

**Figure 7.**
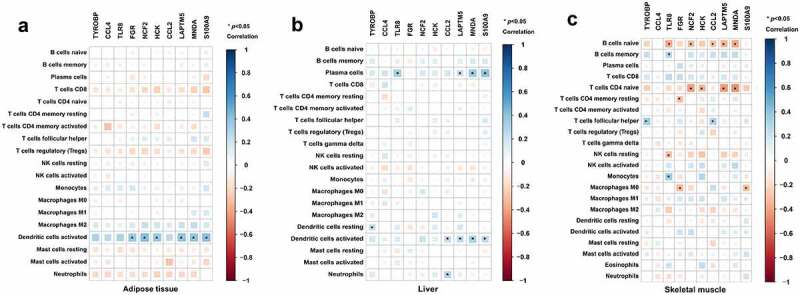
Association of hub genes and infiltrating immune cells in adipose tissue, liver tissue and skeletal muscle. Correlations of hub gene expression and infiltrating immune cells were analysed by Spearman correlation analysis. The heatmaps present correlations of hub gene expression and infiltrating immune cells in (a) adipose tissue, (b) liver tissue and (c) skeletal muscle. *p < 0.05.

Furthermore, a correlation analysis between hub genes and infiltrating immune cells was performed to identify the association of hub genes with immune cell infiltration. As shown in Figure 7a, FGR, NCF2, HCK, LAPTM5, MNDA and S100A9 were positively associated with dendritic cells activated in adipose tissue (*p*< 0.05). As displayed in Figure 7b, TLR8, LAPTM5, MNDA and S100A9 were positively associated with plasma cells; TYROBP was positively associated with resting dendritic cells; CCL2, LAPTM5, MNDA and S100A9 were positively associated with activated dendritic cells; and CCL2 was positively associated with neutrophils in the liver tissue (*p*< 0.05). As in skeletal muscle (Figure 7c), TLR8 was positively associated with memory B cells and monocytes and TYROBP and CCL2 were positively associated with follicular helper T cells (*p* < 0.05). Meanwhile, TLR8, NCF2, CCL2, LAPTM5 and MNDA were negatively associated with naive B cells; NCF2, HCK, LAPTM5 and MNDA were negatively associated with CD4 naive T cells; FGR was negatively associated with resting memory CD4 T cells and M0 macrophages; TLR8 was negatively associated with resting NK cells; and S100A9 was also negatively associated with M0 macrophages (*p* < 0.05, Figure 7c).

### Validation of hub genes

**Figure 8.**
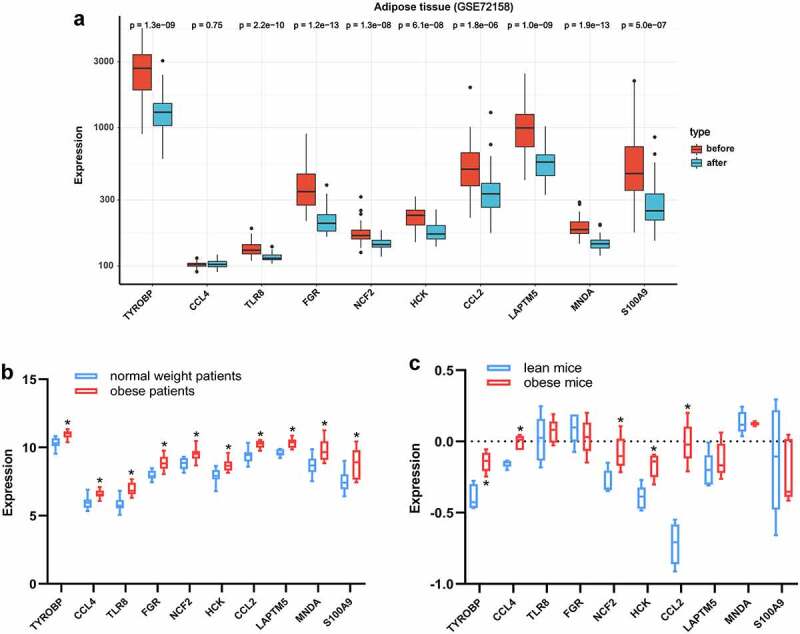
Validation of hub genes in adipose tissue. (a) Expression of hub genes in adipose tissue of obese subjects before (n = 42) and after BS (n = 42) was validated in the GSE72158 dataset. (b) Expression changes of hub genes in adipose tissue between obese (n = 10) and normal weight (n = 10) people were validated in the GSE151839 dataset. (c) Expression changes of hub genes in adipose tissue between obese (n = 5) and lean (n = 5) B6 mice were validated in the Attie Lab Diabetes database. The Wilcoxon test was performed. *p < 0.05.

To validate the expression changes of hub genes after BS, the GSE72158 dataset, which includes data on adipose tissue samples of obese subjects before and after BS was included. Consistent with the results from the bioinformatics analysis, the expression levels of TYROBP, TLR8, FGR, NCF2, HCK, CCL2, LAPTM5, MNDA and S100A9 were all downregulated after BS in the GSE72158 dataset (*p* < 0.05, Figure 8a). The expression changes of the hub genes in adipose tissue were also validated in obese and lean people and mice. As presented in Figure 8b, the expression levels of the ten hub genes were all upregulated in the adipose tissue of obese people (*p* < 0.05). The expression levels of TYROBP, CCL4, NCF2, HCK and CCL2 were also upregulated in the adipose tissue of obese mice (*p* < 0.05, Figure 8c). These results indicated that these hub genes may be pathologically upregulated in obesity and return to normal after BS.

## Discussion

Obesity is increasing, and has become a serious threat to human health. BS is an effective treatment for obesity and associated comorbidities [15]. Adipose tissue, liver tissue and skeletal muscle are key metabolic organs [16]. In the present study, integrative bioinformatic analyses were innovatively performed for these three key metabolic tissues of obese patients before and after BS. We aimed to identify the potential hub genes and pathways that may be closely associated with the beneficial effects of BS.

In our study, in addition to metabolism-related signalling pathways, inflammatory-related signalling pathways were also significantly changed after BS. The GSVA results showed that the IL6-JAK-STAT3 signalling and IL2-STAT5 signalling pathways were downregulated in adipose tissue, liver tissue and skeletal muscle after BS. IL-6 is a proinflammatory factor secreted by T cells, and IL6-JAK-STAT3 signalling is a classical inflammatory pathway in the body [17]. Elevated IL-6 levels in obese patients may have an effect on metabolism and insulin sensitivity [18]. IL-2 is a pleiotropic cytokine, and IL2-JAK-STAT5 signalling can control the differentiation and homoeostasis of both proinflammatory and anti-inflammatory T cells [19]. Kochumon et al. reported that elevated IL-2 expression in obesity correlates with metabolic inflammation and insulin resistance [20]. In accordance with our results, IL-6 was found to be decreased after BS [21]. In addition, a total of 121 common DEGs, which are defined as the intersection of DEGs before and after BS in at least two of the three metabolic tissues, were identified in our study. Uniformly, these common DEGs were also mainly enriched in inflammatory-related and immune-related signalling pathways, such as the neutrophil activation, Toll-like receptor binding, chemokine signalling, IL17 signalling, NF-kappa B signalling pathways. These results indicated that inflammatory-related and immune-related signalling pathways are involved in the effects of BS.

Immune cells play important roles in the metabolic tissues of obese patients. In adipose tissue, there are substantial changes in the amount and function of immune cells in obese patients, leading to adipose tissue dysfunction and the development of obesity-related local and systemic inflammation [22,23]. In liver tissue, macrophages initiate inflammation and CD8^+^ T cells regulate liver injury in obesity [24,25]. NKT cells also play roles in obesity-associated liver disease [11]. Besides, inflammatory macrophage numbers in skeletal muscle are elevated during obesity [26], and there is a positive association between macrophage content and insulin resistance in obesity [27]. CIBERSORT, a method for characterizing the cell composition of complex tissues from their gene expression profiles, has been widely used to evaluate the relative content of 22 kinds of immune cells [28]. Our study also evaluated immune cell infiltration before and after BS in adipose tissue, liver tissue and skeletal muscle by CIBERSORT method. The results showed that significant changes in immune cell infiltration occurred after BS. The abundance of M1 macrophages was significantly lower in adipose and liver tissue after BS. Tissue inflammation is a well-described feature of obesity, and macrophages are key cell types that orchestrate the initiation, specification and resolution of tissue-specific inflammation [29]. M1 macrophages are a subtype of macrophages that are proinflammatory and produce proinflammatory cytokines, such as IL-6 and tumour necrosis factor α [30]. In human obesity, macrophages are polarized towards the M1 type, and weight loss can reduce M1 macrophages [30]. The previous results are in line with our findings, which showed that the number of M1 macrophages was decreased in the adipose tissue of obese subjects after BS [31]. Meanwhile, the abundance of activated dendritic cells was also significantly decreased in adipose tissue after BS. Dendritic cells in adipose tissue were correlated with obesity-associated insulin resistance [32]. Collectively, these results demonstrated that the changes in immune cell infiltration after BS may also be involved in the effects of BS. In accordance with our findings, several changes in adaptive immunity after BS have also been previously reported, including a reduction in CD4 and CD8 T-cell counts and an increase in B regulatory cells [13].

By constructing a PPI network and using the degree method in the CytoHubba analysis, ten hub genes were identified: TYROBP, CCL4, TLR8, FGR, NCF2, HCK, CCL2, LAPTM5, MNDA and S100A9. These ten hub genes were all downregulated after BS in the three key metabolic tissues. Moreover, our results verified that the expression levels of the 10 hub genes were all increased in obese people. Therefore, BS may alleviate obesity by decreasing the expression of these hub genes. Abundant evidence has confirmed that TYROBP is a key regulator of the immune system and plays important roles in signal transduction in dendritic cells, monocytes and macrophages [33]. CCL4 is a chemoattractant for critical immune regulatory cells such as monocytes, macrophages and dendritic cells [34]. CCL2, also known as monocyte chemoattractant protein-1, can also promote cell migration [35]. Elevated CCL4 and CCL2 expression levels were reported in obesity and found to be correlated with inflammation and insulin resistance [34–36]. TLR8 is an important pattern recognition receptor that acts as the main bridge between innate immunity and specific immunity [37]. FGR is required for proinflammatory macrophage activation during diet-induced obesity [38]. LAPTM5, which is preferentially expressed in immune cells, is a positive regulator of proinflammatory signalling pathways in macrophages [39]. In addition, S100A9, which is constitutively expressed in neutrophils and monocytes, was significantly increased in obese diabetes patients, and the increased levels of S100A9 were related to the macrophage content in obesity [40]. Taken together, these hub genes are mainly involved in the regulation of immune cells and inflammation.

Importantly, these hub genes were also found to be associated with infiltrating immune cells in our study. For example, TYROBP was positively associated with resting dendritic cells in the liver tissue and with follicular helper T cells in skeletal muscle. TLR8 was positively associated with plasma cells in the liver tissue, and with memory B cells and monocytes in skeletal muscle but negatively associated with naive B cells and resting NK cells in skeletal muscle. FGR was positively associated with activated dendritic cells in adipose tissue but negatively associated with resting memory CD4 T cells and M0 macrophages in skeletal muscle. LAPTM5, MNDA and S100A9 were positively associated with activated dendritic cells in adipose tissue and positively associated with plasma cells and activated dendritic cells in the liver tissue. Therefore, BS may influence immune cell infiltration in metabolic tissues by regulating the expression of these hub genes, thereby reducing weight and improving metabolism.

Certain limitations were observed in our present study. First, this is a retrospective analysis of public databases, and sufficient demographic and clinical information that may influence the immune regulation mechanism of BS was absent. Second, the follow-up times of these datasets in our study were different. GSE59034 and GSE66921 were followed up for 2 years, GSE106737 and GSE5109 were followed up for 1 year, and GSE134913 was followed up for 2 weeks and 52 weeks. Therefore, further experimental verification is still needed to confirm our findings.

In conclusion, this study innovatively combined the results of three important metabolic tissues, adipose tissue, liver tissue and skeletal muscle, performed integrative bioinformatic analyses to analyse the potential immune and inflammatory mechanisms of BS, and revealed the hub genes (TYROBP, TLR8, FGR, NCF2, HCK, CCL2, LAPTM5, MNDA and S100A9) associated with changes in immune cell infiltration after BS. The target miRNAs and TFs of these hub genes were analysed, and potential therapeutic drugs were also predicted. These results helped to clarify the mechanisms of BS and provided evidence for potential nonsurgical therapies for obesity by improving immune cell infiltration and inflammation. Further studies are still needed to verify and reveal further mechanisms.

## Supplementary Material

Supplemental Material

## Data Availability

The data that support the findings of this study are available in the GEO database (http://www.ncbi.nlm.nih.gov/geo) under reference numbers [GSE59034, GSE66921, GSE106737, GSE134913, GSE5109, GSE72158, and GSE151839].
