# Supplementary figures and images for: Integrative analyses of hub genes and their association with immune infiltration in adipose tissue, liver tissue and skeletal muscle of obese patients after bariatric surgery
Source: Adipocyte. 2022 Apr 12;11(1):190–201. doi: 10.1080/21623945.2022.2060059 (PMC9009953; doi:10.1080/21623945.2022.2060059)

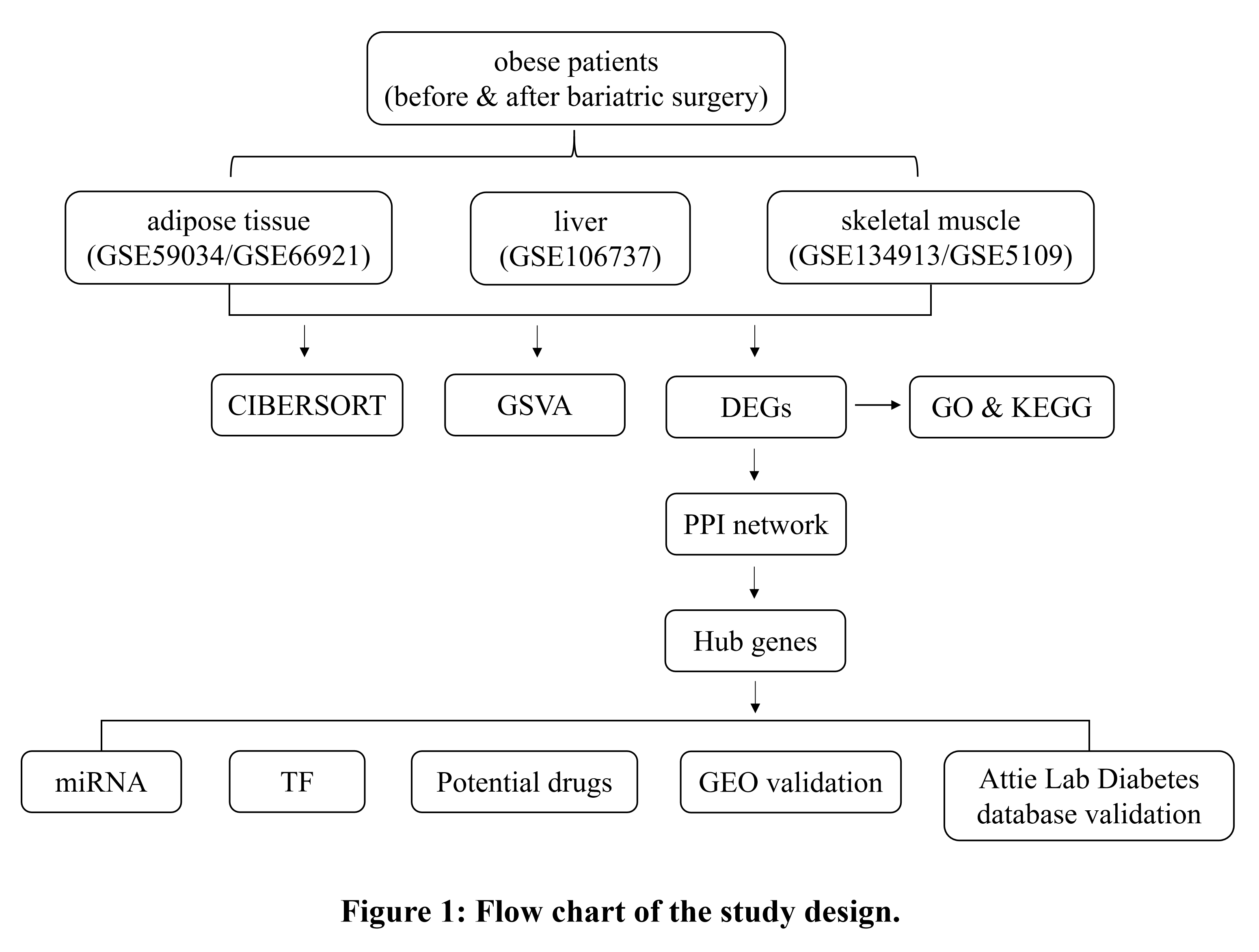

Supplement: Supplemental Material [file KADI_A_2060059_SM0410.zip › supplementary/Supplemental Figure 1.tif]
